# Chiral gold(I) vs chiral silver complexes as catalysts for the enantioselective synthesis of the second generation GSK-hepatitis C virus inhibitor

**DOI:** 10.3762/bjoc.7.111

**Published:** 2011-07-19

**Authors:** María Martín-Rodríguez, Carmen Nájera, José M Sansano, Abel de Cózar, Fernando P Cossío

**Affiliations:** 1 Departamento de Química Orgánica e Instituto de Síntesis Orgánica, Universidad de Alicante, Apdo. 99, E-03080 Alicante, Spain; 2 Departamento de Química Orgánica I, Facultad de Química, Universidad del País Vasco, Apdo. 1072, E-20018 San Sebastián, Spain

**Keywords:** BINAP, 1,3-dipolar cycloaddition, gold, HCV, phosphoramidite, silver, viral inhibitor

## Abstract

The synthesis of a GSK 2^nd^ generation inhibitor of the hepatitis C virus, by enantioselective 1,3-dipolar cycloaddition between a leucine derived iminoester and *tert*-butyl acrylate, was studied. The comparison between silver(I) and gold(I) catalysts in this reaction was established by working with chiral phosphoramidites or with chiral BINAP. The best reaction conditions were used for the total synthesis of the hepatitis C virus inhibitor by a four step procedure affording this product in 99% ee and in 63% overall yield. The origin of the enantioselectivity of the chiral gold(I) catalyst was justified according to DFT calculations, the stabilizing coulombic interaction between the nitrogen atom of the thiazole moiety and one of the gold atoms being crucial.

## Introduction

**Figure 1:**
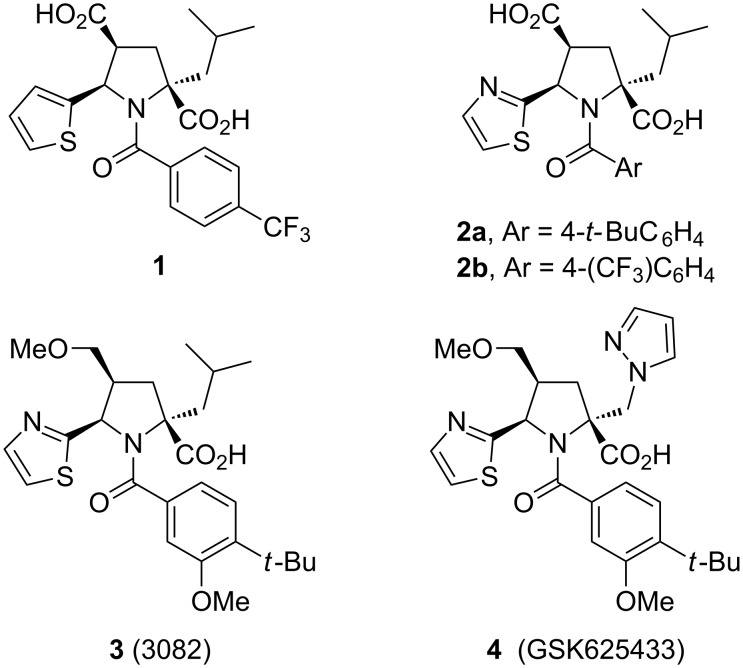
More active GSK HCV inhibitors.

The prevalence of chronic hepatitis C virus (HCV) infection is such that it is estimated to be suffered by around 200 million people worldwide [1]. This enveloped single-stranded RNA virus (belonging to the *Flaviviridae* family) is present in six major genotypes in the world’s industrialized nations, genotype 1 being the most prevalent, followed by genotype 2 and 3. Due to the poor toleration of the current therapy, and the lack of an appropriate vaccine, researchers working on strategies for developing antivirals have tried to attack viruses at every stage of their life cycles, namely attachment to a host cell, replication of viral components, assembly of viral components into complete viral particles and release of viral particles able to infect new hosts cells. Inside the infected hepatocytes, structural E1 and E2 and non-structural proteins such as NS2, NS3 (which bear serine proteinase, helicase, and NTPase activities), NS4A, NS4B, NS5A (regulators of RNA replication), and NS5B (the RNA-dependent RNA polymerase) are generated [2–3] and, in fact, constitute the main targets. At the moment, there are many drugs under clinical trial evaluation, the compounds targeting HCV replication being the most promising candidates to achieve a sustained virological response [1,4]. Several years ago, a high-throughput screening of the GlaxoSmithKline compound collection identified a series of small pyrrolidine molecules, e.g., **1** (Figure 1), able to inhibit the RNA-dependent RNA polymerase of the virus responsible for hepatitis C (genotype 1g) [5]. Thus, their high replication rates (billions of copies per day) can be drastically suppressed by the inhibition of the NS5B RNA-dependent RNA polymerase enzyme, which is the primary target for oral antiviral agents [6–7]. In further studies, a second generation of antiviral agents **2** and **3** (Figure 1), offering a greater dynamic range even for HCV genotype 1b, was published [5,8–9]. These molecules incorporated a 2-thiazole heterocycle instead of the 2-thienyl group, together with a more hydrophobic environment at the amido group [9–12]. However, the design of improved broader spectrum compounds, capable of effective inhibition of genotypes 1a and 1b, is desirable. In this sense, GSK625433 (**4**) (Figure 1) has exhibited a good pharmacokinetic profile in preclinical animal species [13].

**Scheme 1:**
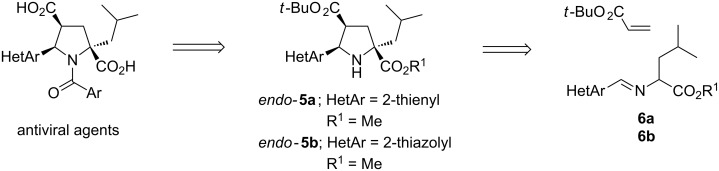
Retrosynthetic analysis of antiviral structures.

The synthesis of the *endo-*pyrrolidine core of **5** is the key step for the preparation of these antiviral agents, and can be efficiently achieved by a 1,3-dipolar cycloaddition (1,3-DC) between the corresponding azomethine ylide and an alkyl acrylate [14–18] (Scheme 1). The first synthesis of racemic product **1**, and other derivatives including compounds **2**, was achieved in several steps using, as the key reaction, the silver(I) or lithium(I)-metalloazomethine ylide, under basic conditions, and *tert-*butyl acrylate. The enantiomeric samples were isolated by semi-preparative chiral HPLC [9–10]. The first *endo*-diastereoselective synthesis of the key precursor **5a** (HetAr = 2-thienyl), of the antiviral agent **1** (96% de), was achieved by our group from imine **6a** (HetAr = 2-thienyl; R^1^ = Me) in the presence of the acrylate derived from (*R*)-methyl lactate [19]. However, the most straightforward, and also faster, approach to the enantiomeric formation of this non-nucleosidic antiviral agent **1** is based on a catalytic enantioselective 1,3-DC [20–24]. The first reported enantioselective overall synthesis of the structure **1** was catalyzed by a chiral phosphoramidite and AgClO_4_ [25–26], although the synthesis of the five-membered core has also been published using chiral calcium complexes [27–28].

In addition, for the second generation antivirals **2** or **3**, the efficiency of the Lewis acid-catalyzed 1,3-DC, following the route shown in Scheme 1, was combined with hydroquinine as chiral base (6 mol %) together with silver acetate (3 mol %), and this afforded moderate enantioselectivities (70–74%) of **5b**, in such a way that a further 1,1’-binaphthyl-2,2’-dihydrogen phosphate assisted chiral resolution was required to increase the optical purity of the target molecule [11]. Chiral calcium(II) complexes have been used for the synthesis of a similar key molecule **5b** (R^1^ = *t*-Bu, 88% ee), but the overall synthesis of the antiviral drug was not reported [27–28].

In this article, we describe the full study concerning the enantioselective synthesis of product **5b** using silver(I) or gold(I) complexes, generated from chiral phosphoramidites or BINAP as ligands, in order to prepare antiviral agent **2a**.

## Results and Discussion

The efficiency of the chiral phosphoramidite/silver(I) salts [25–26,29] and BINAP/Ag(I) salts [30–31] in 1,3-DC, following the general pattern shown in Scheme 1, has been demonstrated by our group, establishing a wider scope and sensibly higher enantioselectivities for the reactions performed in the presence of chiral phosphoramidite/silver(I) complexes [24]. Concerning enantioselective gold(I)-catalyzed 1,3-DC, the classical cycloaddition starting from iminoesters **6** has not been so extensively explored. Reports of chiral transformations involving azlactones [32–33] and iminoesters **6** [34], which employed chiral diphosphines and gold(I) salts, have been published showing very good *endo*-diastereoselectivities and moderate to excellent enantioselectivities. However, the use of acrylates as dipolarophiles has only been explored with the 2-thienyliminoesters **6a**.

**Figure 2:**
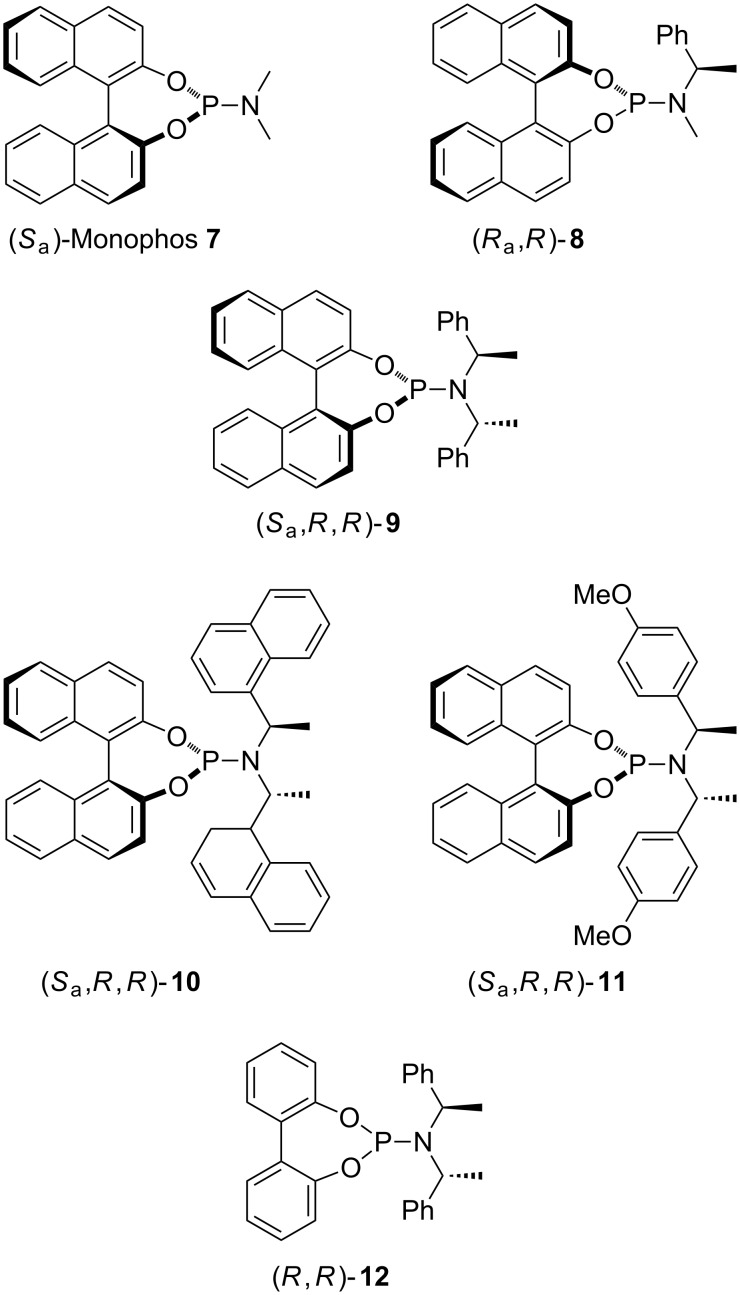
Chiral phosphoramidites tested in this study.

Therefore, based on our experience of silver(I)- and gold(I)-catalyzed 1,3-DC involving azomethine ylides derived from α-iminoester **6b** and *tert-*butyl acrylate, we selected a series of known chiral phosphoramidite ligands (Figure 2), which were prepared according to the literature [35]. The chiral phosphoramidite/silver(I) complexes were generated in situ by mixing equimolar amounts of both components at room temperature for 30 min. Chiral phosphoramidite/AuCl complexes were generated according to the literature [36] and, finally, underwent anion interchange in the presence of the corresponding silver salt. The precipitate was filtered through a celite pad and used without any other additional treatment.

**Scheme 2:**
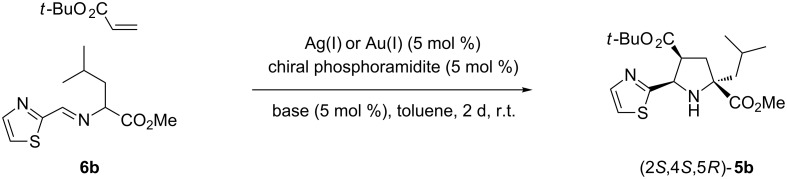
Optimization of the reaction conditions for the synthesis of the key intermediate **5b**.

All of the reactions were performed at room temperature, employing a 5 mol % of both catalyst and base, for 17 h (Scheme 1). Reactions between iminoester **6b** and *tert*-butyl acrylate, which employed silver complexes derived from Monophos (*S*_a_)-**7** ligand, afforded racemic *endo*-cycloadduct **5b** (Table 1, entries 1–3). The analogous reaction catalyzed by chiral phosphoramidite **7**/gold(I) complexes did not occur at all when AgClO_4_ or AgSbF_6_ were employed as anion interchange agents. Just a small conversion, with some side products, and null enantioselectivity was observed in the crude reaction mixtures obtained when using (*S*_a_)-**7**/AuTFA (Table 1, entry 4). When the reaction was carried out in the presence of chiral ligand (*R*_a_,*R*)-**8** the enantioselectivities were low or moderate in the examples concerning AgClO_4_ and AgTFA (TFA = trifluoroacetate anion), respectively (Table 1, entries 5 and 7). Surprisingly, the reaction involving this chiral ligand **8** combined with AgSbF_6_ afforded a good yield of the enantiomerically pure cycloadduct **5b** (Table 1, entry 6). Attempts to increase the enantioselectivity, in the example run with AgTFA, by replacing triethylamine by diisopropylethylamine (DIPEA) were not successful, and only a slight increment of enantiomeric excess was observed (Table 1, entry 8). Again, the gold complex (*R*_a_,*R*)-**8**/AuTFA did not give the expected reaction product (Table 1, entry 9). The employment of this matched combination with (*R*_a_,*R*)-**8** was justified by the low enantioselectivity achieved through the use of (*R*_a_,S)-**8** in the same transformation (not shown in Table 1). The widely used chiral ligand (*S*_a_,*R*,*R*)-**9** has also been similarly studied. In this case, the matched combination was determined in previous works that investigated the scope of enantioselective silver(I)-catalyzed 1,3-DC of azomethine ylides and dipolarophiles [25,29]. The enantioselectivities were moderate, even when using AgSbF_6_, and the effect of the added base was negligible (Table 1, entries 10–14). The process catalyzed by the (*S*_a_,*R*,*R*)-**9**/AuTFA was not suitable (Table 1, entry 15). The more sterically hindered chiral phosphoramidite (*S*_a_,*R*,*R*)-**10** did not afford any interesting results because the conversions were extremely low after 2 days reaction, and the crude reaction mixture was very complex (^1^H NMR analysis) (Table 1, entries 16–18). However, a good result was obtained when phosphoramidite (*S*_a_,*R*,*R*)-**11** was tested together with AgClO_4_. The high enantioselectivity achieved for **5b** (86% ee) is in contrast to the racemic samples identified when either AgSbF_6_ or AgTFA were employed as co-catalysts (Table 1, entries 19–21). Biphenol derived ligand (*R*,*R*)-**12** generally furnished good yields of the cycloadduct **5b** but with a low enantiodiscrimination (Table 1, entries 22–24). In many examples, although the reactions were performed at lower temperatures (0 or −20 °C, not shown in Table 1) the resulting enantioselectivities did not suffer noticeable variations. In all of the cases given in Table 1, the *endo*-cycloadduct was exclusively generated, and the absolute configuration of **5b** was established by extrapolation with the results previously obtained for each chiral catalyst [25–26,28–30,33]. According to these results the combination of chiral phosphoramidite and silver(I) salt is much more appropriate than the analogous one made with gold(I) salts. Especially useful is the reaction of (*R*_a_,*R*)-**8**/AgSbF_6_ catalytic complex affording enantiomerically pure cycloadduct *endo*-**5b**. It is worth mentioning that chiral phosphoramidite/gold(I) complexes, formed by anion interchange of the corresponding phosphoramidite/AuCl complex and AgSbF_6_ [36] or AgBF_4_ [37–38], have been successfully employed in enantioselective cycloaddition of allenedienes [36–37] or allenenes [38] under very mild reaction conditions (0 ºC to r.t.). Despite these described opportunities provided by chiral phosphoramidite ligands as a part of gold(I) complexes, their activity (see Table 1) was negligible, until now, when applied in the 1,3-DC represented in Scheme 2.

**Table 1:** Optimization of the 1,3-dipolar cycloaddition of **6b** and *tert-*butyl acrylate using chiral phosphoramidite ligands.

Entry	Catalyst^a^	Base	Yield^b^ (%)	ee^c^ (%)

1	(*S*_a_)-**7**/AgClO_4_	Et_3_N	^___d^	*rac*
2	(*S*_a_)-**7**/AgSbF_6_	Et_3_N	^___d^	*rac*
3	(*S*_a_)-**7**/AgTFA	Et_3_N	^___d^	*rac*
4	(*S*_a_)-**7**/AuTFA	DIPEA	^___d^	^___d^
5	(*S*_a_,*R*)-**8**/AgClO_4_	Et_3_N	82	20
6	(*S*_a_,*R*)-**8**/AgSbF_6_	Et_3_N	82	99
7	(*S*_a_,*R*)-**8**/AgTFA	Et_3_N	82	60
8	(*S*_a_,*R*)-**8**/AgTFA	DIPEA	82	64
9	(*S*_a_,*R*)-**8**/AuTFA	DIPEA	^___d^	^___d^
10	(*S*_a_,*R*,*R*)-**9**/AgClO_4_	DIPEA	86	30
11	(*S*_a_,*R*,*R*)-**9**/AgSbF_6_	Et_3_N	72	40
12	(*S*_a_,*R*,*R*)-**9**/AgSbF_6_	DIPEA	82	40
13	(*S*_a_,*R*,*R*)-**9**/AgTFA	Et_3_N	82	50
14	(*S*_a_,*R*,*R*)-**9**/AgTFA	DIPEA	82	40
15	(*S*_a_,*R*,*R*)-**9**/AuTFA	DIPEA	^___d^	^___d^
16	(*S*_a_,*R*,*R*)-**10**/AgClO_4_	Et_3_N	^___d^	*rac*
17	(*S*_a_,*R*,*R*)-**10**/AgTFA	Et_3_N	^___d^	*rac*
18	(*S*_a_,*R*,*R*)-**10**/AgSbF_6_	Et_3_N	^___d^	*rac*
19	(*S*_a_,*R*,*R*)-**11**/AgClO_4_	Et_3_N	72	86
20	(*S*_a_,*R*,*R*)-**11**/AgSbF_6_	Et_3_N	^___d^	*rac*
21	(*S*_a_,*R*,*R*)-**11**/AgTFA	Et_3_N	^___d^	*rac*
22	(*R*,*R*)-**12**/AgClO_4_	Et_3_N	79	30
23	(*R*,*R*)-**12**/AgTFA	Et_3_N	87	40
24	(*R*,*R*)-**12**/AgSbF_6_	Et_3_N	86	30

^a^The generation of silver catalysts was achieved by mixing equimolar amounts of silver(I) or gold(I) salt and the corresponding phosphoramidite. ^b^After flash chromatography (silica gel). The observed *endo*:*exo* ratio was always >98:2 (^1^H NMR). ^c^Determined by using analytical chiral HPLC columns (Daicel, Chiralpak AS). ^d^Not determined.

**Scheme 3:**
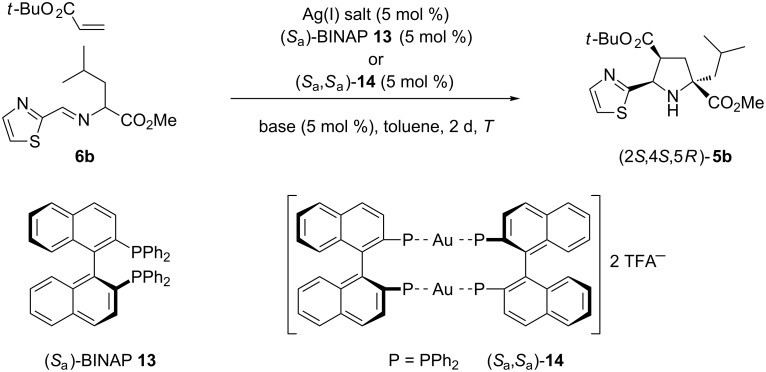
Preparation of the enantiomerically enriched **5b**.

The chiral ligand (*S*_a_)-BINAP (**13**) was also tested in the standard reaction to access key molecule *endo-***5b** (Scheme 3). AgClO_4_ was found to be the most appropriate silver salt to achieve the highest enantioselectivity (88% ee) compared to the results obtained when other silver salts were employed (Table 2, entries 1, 3, and 4). In agreement with the previous results, the reaction with chiral silver complexes at lower temperatures did not improve the enantioselectivity. According to our previous work, dimeric chiral gold(I) catalyst [(*S*_a_)-BINAPAuTFA]_2_ (*S*_a_,*S*_a_)-**14** was very efficient in 1,3-DC compared to other catalysts with different stoichiometry or anion nature. The gold complex (*S*_a_,*S*_a_)-**14** was prepared according to the literature [39] and immediately used in the cycloaddition in the absence of base because of its bifunctional behaviour, namely the activation of the basic character of the dipole [34]. However, no reaction occurred under these conditions (Table 2, entry 5). Therefore, the presence of the base was crucial for the evolution of the reaction, as can be seen in entries 6 and 7 of Table 2. Triethylamine promoted the reaction affording good yield and good enantioselectivity (78% ee). However, DIPEA-mediated cycloaddition did not improve the enantioselectivity of the resulting *endo*-cycloadduct **5b**. Unlike the results obtained with silver(I) catalytic complexes at lower temperatures (0 or −20 °C), the gold(I)-catalyzed cycloaddition could be successfully carried out at 0 °C resulting in excellent enantiodiscrimination (99% ee) to the detriment of the reaction time, which had to be increased to 3 days (Table 2, entry 8). The result obtained in this last example was excellent but the enantiomeric excess achieved at room temperature in the reaction performed with (*S*_a_)-**13**/AgClO_4_ complex is also valuable.

**Table 2:** Optimization of the 1,3-dipolar cycloaddition of **6a** and *tert-*butyl acrylate using chiral (*S*_a_)-BINAP (**13**) ligand.

Entry	Catalyst^a^	Base	Yield^b^ (%)	ee^c^ (%)

1	(*S*_a_)-**13**/AgClO_4_	Et_3_N	78	88
2	(*S*_a_)-**13**/AgClO_4_	Et_3_N^d^	75	85
3	(*S*_a_)-**13**/AgSbF_6_	Et_3_N	79	72
4	(*S*_a_)-**13**/AgTFA	Et_3_N	82	40
5	(*S*_a_,*S*_a_)-**14**	**^___^**	^___e^	^___e^
6	(*S*_a_,*S*_a_)-**14**	Et_3_N	90	78
7	(*S*_a_,*S*_a_)-**14**	DIPEA	87	70
8	(*S*_a_,*S*_a_)-**14**	Et_3_N^d,f^	92	99

^a^The generation of silver catalysts was achieved by mixing equimolar amounts of silver(I) and (*S*_a_)-BINAP. ^b^After flash chromatography (silica gel). The observed *endo*:*exo* ratio was always >98:2 (^1^H NMR). ^c^Determined using analytical chiral HPLC columns (Daicel, Chiralpak AS). ^d^Reaction performed at 0 °C. ^e^Not determined. ^f^After 3 days reaction.

**Scheme 4:**
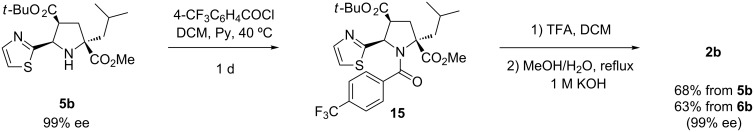
Total synthesis of antiviral agent **2b**.

With the most enantiomerically enriched cycloadduct **5b**, the synthesis of the antiviral agent **2a** could be accomplished in two conventional steps involving an amidation reaction and a double ester hydrolysis. The latter step consisted of a first stage TFA-mediated hydrolysis of the *tert*-butyl ester followed by a basic stage employing a refluxing solution of KOH/MeOH (Scheme 4). The final product **2b** was finally isolated in 68% overall yield (from pyrrolidine **5b**) and with 99% ee, or alternatively in 63% overall yield from iminoester **6b**.

**Figure 3:**
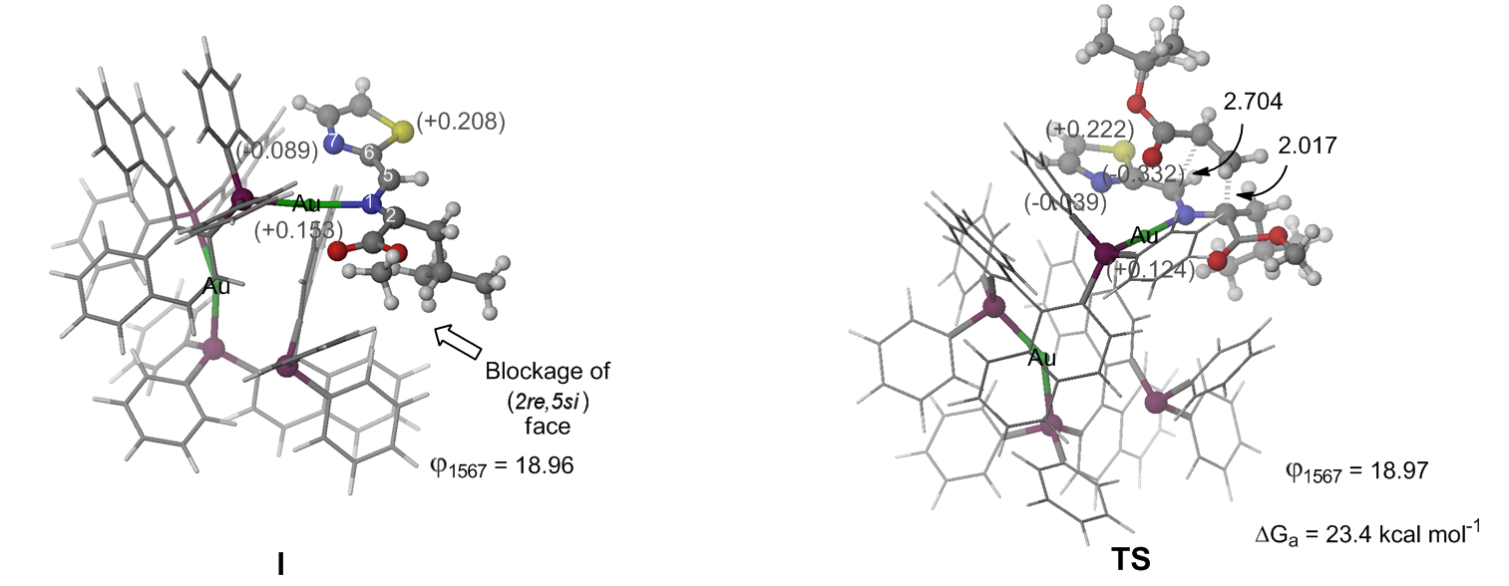
Gibbs activation energy and main geometrical features of the computed ylide and transition structures (TS) corresponding to the 1,3-DC of the Au(I)–ylide complex and *tert*-butyl acrylate computed at ONIOM(B3LYP/LanL2DZ:UFF) level of theory. High-level and low-level layers are represented as ball & stick and wireframe models, respectively. Grey numbers in parentheses represent Mulliken charges. Distances are in Å.

Although the study of the enantioselectivity exhibited by chiral phosphoramidite/silver(I) complexes employing DFT calculations was confirmed by our group [25], an explanation for the excellent results obtained employing the gold complex (*S*_a_,*S*_a_)-**14** (Table 2, entry 8) was needed. In a previous work, we demonstrated that the stereoselectivity of the 1,3-DC employing chiral metallic Lewis bases arises from the blockage of one of the prochiral faces [40]. In this way, our results (in terms of DFT calculations) show that there is only one energetically accessible conformation due to the high substitution of the leucine-derived ylide (Figure 3). In this reactive complex there is an effective blockage of the (2*re*,5*si*) prochiral face of the ylide. Therefore, the predicted stereochemical outcome corresponds to the exclusive formation of the (2*S*,4*S*,5*R*)-**5b** cycloadduct, the same as that obtained experimentally.

As shown in Figure 3, the reaction proceeds to a concerted but highly asynchronous cycloaddition in which the *endo*-approach of the dipolarophile is favoured due to a stabilizing interaction of the carboxylic group and the metallic centre. The computed activation Gibbs free energy barrier associated with the formation of (2*S*,4*S*,5*R*)-**5b** is 23.4 kcal mol^−1^, which means that the process is feasible at the reaction temperature. It is worth noting that there is a stabilizing coulombic interaction between the nitrogen atom of the thiazole moiety (N_7_) and one of the gold atoms of the catalyst, both in the TS and the ylide complex. This interaction fixes the planar conformation of the ylide moiety and minimizes the possible steric hindrance with the bulky *tert*-butyl group of the dipolarophile. When a phenyl substituent is placed to the imino group this planar conformation does not exist and, in consequence, a more steric interaction avoids the approach of the mentioned dipolarophile.

## Conclusion

In this work the complexity of the 1,3-DC reaction of azomethine ylides and dipolarophiles (in this case acrylates) was demonstrated. There are many parameters to control and a small variation can cause a dramatic effect in the overall enantiodiscrimination of the process. The temperature does not equally affect silver(I) and gold(I) catalysts. The effect of the heterocycle remains crucial in these transformations because, originally, the enantioselectivity of the reaction between methyl benzylideneiminoglycinate and alkyl acrylates failed in the presence of the silver(I) or the dimeric gold(I) complexes derived from chiral BINAP. The metal cation and the counterion are also important in the final result and, in certain cases, their position with respect to the reaction centre can modify the overall reaction and consequently alter the enantioselectivity of the process. To date, the best reaction conditions to access GSK 2^nd^ generation antiviral drugs **2a** are: The employment of chiral phosphoramidite (*R*_a_,*R*)-**8**/AgSbF_6_ and Et_3_N (both in 5 mol % amount) at r.t. for 2 h, or chiral (*S*_a_,*S*_a_)-**14** gold complex and Et_3_N (both in 5 mol % amount) at 0 °C for 3 days. Whilst phosphoramidite complexes operated exclusively in the presence of silver salts, the most versatile chiral BINAP ligand could work efficiently with both silver(I) or gold(I) cations. The stabilizing coulombic interaction between the nitrogen atom of the thiazole moiety and one of the gold atoms of the catalyst both in the TS and the ylide complex is the explanation for the success of the gold-catalyzed cycloaddition, in constrast to the observed TS involving methyl benzylideneiminoleucinate.

## Experimental

**General**. All reactions were carried out in the absence of light. Anhydrous solvents were freshly distilled under an argon atmosphere. Aldehydes were also distilled prior to use for the elaboration of the iminoesters. Melting points were determined with a Reichert Thermovar hot plate apparatus and are uncorrected. Only the structurally most important peaks of the IR spectra (recorded on a Nicolet 510 P-FT and on a Jasco FTIR 4100) are listed. ^1^H NMR (300 MHz) and ^13^C NMR (75 MHz) spectra were obtained on a Bruker AC-300 using CDCl_3_ as solvent and TMS as internal standard, unless otherwise stated. Optical rotations were measured on a Perkin Elmer 341 polarimeter. HPLC analyses were performed on a JASCO 2000-series equipped with a chiral column (detailed for each compound in the main text), using mixtures of *n*-hexane/isopropyl alcohol as mobile phase, at 25 °C. Low-resolution electron impact (EI) mass spectra were obtained at 70 eV on a Shimadzu QP-5000 and high-resolution mass spectra were obtained on a Finnigan VG Platform. HRMS (EI) were recorded on a Finnigan MAT 95S. Microanalyses were performed on a Perkin Elmer 2400 and a Carlo Erba EA1108. Analytical TLC was performed on Schleicher & Schuell F1400/LS silica gel plates and the spots were visualized under UV light (λ = 254 nm). For flash chromatography we employed Merck silica gel 60 (0.040–0.063 mm). Ligands **7**–**12** were prepared according to the reported procedure (see text). All of the transformations performed with silver catalysts were performed in the absence of light. The synthesis of the already characterized chiral complex (*S*_a_,*S*_a_)-**14** was performed according to the published procedure [39].

**Computational methods.** Hybrid QM/MM calculations for optimizations of saddle points were performed in terms of ONIOM [41–43] method implemented in GAUSSIAN09 suite of programs [44]. Ball & stick model in Figure 3 shows atoms included in the high-level layer, and a wire model is used to represent atoms included in the low-level layer. In the high-level layer, the electron correlation was partially taken into account by using the hybrid functional B3LYP [45–50] combined with Hay-Wadt small core effective potential (ECP) [51] basis set. UFF [52] molecular mechanics force field was employed in the low-level layer. Thermal corrections of Gibbs free energies were computed at the same level of theory and were not scaled. All stationary points were characterized by harmonic analysis. Reactant intermediates and cycloadducts have positive definite Hessian matrices. Transition structures show only one negative eigenvalue in their diagonalized force constant matrices, and their associated eigenvectors were confirmed to correspond to the motion along the reaction coordinate under consideration.

**1,3-Dipolar cycloaddition of iminoester 6b and *tert-*butyl acrylate. General procedure.** To a solution of the in situ prepared chiral gold complex or chiral silver complex (0.05 mmol) in toluene (2 mL) was added, at r.t., a solution of the iminoester **6b** (120 mg, 0.5 mmol) and *tert-*butyl acrylate (109 μL, 0.75 mmol) in toluene (2 mL). In some cases DIPEA or triethylamine (0.05 mmol) was added (see Tables) and the mixture stirred at r.t. or 0 °C for 2 or 3 days (see Tables). The reaction mixture was filtered off through a celite pad, the organic filtrate was directly evaporated and the residue was purified by recrystallization or by flash chromatography, yielding pure *endo-*cycloadduct **5b**.

(2*S*,4*S*,5*R*)-4-*tert*-Butyl-2-methyl-2-isobutyl-5-(thiazol-2-yl)pyrrolidine-2,4-dicarboxylate (**5b**): Colourless solid; mp >195 °C dec (*n*-hexane/ethyl acetate); [α]_D_^20^ +43 (*c* 1.00, CH_2_Cl_2_, 99% ee by HPLC); IR (neat) ν_max_: 3330, 1718 cm^−1^; ^1^H NMR (300 MHz, CDCl_3_) δ 7.70, 7.27 (2 × d, *J* = 3.4 Hz, 2H, C*H*C*H*S), 4.83 (d, *J* = 7.5 Hz, 1H, CHCS), 3.73 (s, 3H, OCH_3_), 3.41 (q, *J* = 7.8 Hz, 1H, C*H*CHN), 3.25 (br. s, 1H, NH), 2.79, 2.10 (2 × dd, *J* = 13.5, 7.9 Hz, 2H, CH_2_CCO), 1.76–1.69 (m, 2H, C*H*_2_CH), 1.54–1.48 (m, 1H, CH_2_C*H*), 1.17 (s, 9H, (CH_3_)_3_), 0.94, 0.85 (2 × d, *J* = 6.2 Hz, 6H, 2 × CH_3_C); ^13^C NMR (75 MHz, CDCl_3_) δ 176.2, 170.8, 170.6 (2 × CO and CSN), 142.4, 118.8 (CHCHS), 80.6 (*C*(CH_3_)_3_), 68.3 (CO*C*N), 61.7 (*C*HCS), 52.2 (OCH_3_), 49.6 (*C*HCO), 49.3 (*C*H_2_CCO), 39.5 (*C*H_2_CH), 27.6 ((CH_3_)_3_), 25.0 (*C*(CH_3_)_2_), 24.3, 22.9 (2 × *C*H_3_C); EIMS *m/z* (% relative intensity): 368 (M^+^, 1), 310 (51), 295 (16), 255 (23), 254 (14), 253 (100); HRMS calcd for C_18_H_28_N_2_O_4_S, 368.1770; found, 368.1761; HPLC (Chiralpak AD-H), *n*-hexane:iPrOH 95/5, 1 mL/min, λ = 225 nm, *t*_R,maj_ = 12 min, *t*_R,min_ = 18 min.

**Synthesis of the antiviral agent 2b.** Compound (2*S*,4*S*,5*R*)*-***5b** (1.2 mmol, 441 mg) was dissolved in dichloromethane (25 mL), and pyridine (2.4 mmol, 174 μL) and 4-(trifluoromethyl)benzoyl chloride (1.2 mmol, 182 μL) were slowly added at 0 °C. The resulting mixture was refluxed for 1 day and the solvent was removed under vacuo (15 Torr). Crude compound (2*S*,4*S*,5*R*)*-***15**, was allowed to react with trifluoroacetic acid/dichloromethane mixture (9.6 mL/18 mL). The resulting mixture was stirred at r.t. overnight and the solvent evaporated under vacuo. The residue was dissolved in a 1 M solution of KOH in a 4/1 MeOH/H_2_O (50 mL) and refluxed for 16 h. Methanol was evaporated and aqueous HCl (0.5 M, 20 mL) and ethyl acetate were added (2 × 20 mL). The combined organic phases were dried (MgSO_4_) and evaporated, yielding the crude compound (2*S*,4*S*,5*R*)*-***2b**, which was recrystallized from a mixture containing *n*-hexane/ethyl acetate.

(2*S*,4*S*,5*R*)-2-Isobutyl-5-(thiazol-2-yl)-1-[4-(trifluoromethyl)benzoyl]pyrrolidine-2,4-dicarboxylic acid (**2b**): Pale brown solid; mp >130 °C dec (*n*-hexane/ethyl acetate); [α]_D_^20^ +35 (*c* 0.3, toluene, 99% ee); IR (neat) ν_max_: 3100, 1731, 1693 cm^−1^; ^1^H NMR (300 MHz, CD_3_COCD_3_) δ 8.15 (d, *J* = 7.5 Hz, 2H, ArH), 7.80–7.64 (m, 3H, ArH and C*H*CHS), 7.29 (d, *J* = 3.4 Hz, 1H, CHC*H*S), 5.85 (d, *J* = 8.7 Hz, 1H, CHNS), 4.01–3.81 (m, 1H, CHCO), 2.84 (t, *J* = 13.3 Hz, 1H, CH_2_CCO), 2.34 (dd, *J* = 13.2, 6.5 Hz, 1H, CH_2_CCO), 1.28 (m, 4H, C*H*_2_CH and 2 × OH), 1.14–1.06 (m, 1H, CH_2_C*H*), 0.85 (m, 6H, 2 × CH_3_C); ^13^C NMR (75 MHz, CDCl_3_) δ 172.4, 169.1, 168.9, 167.4 (3 × CO and CNS), 141.1, 134.2, 134.1, 130.2, 126.9, 125.4, 120.9, (ArC, CF_3_, and CHCHS), 69.7 (CO*C*N), 65.3 (N*C*H), 51.39 (CHCO), 42.3 (CH_2_CCO), 35.3 (*C*H_2_CH), 25.7 (*C*H(CH_3_)_2_), 24.4, 24.2 (CH(*C*H_3_)_2_); ESIMS *m/z* (% relative intensity) 470 (M^+^, 2); HRMS calcd for C_21_H_21_F_3_N_2_O_5_S, 470.4620; found, 470,4631; HPLC (Chiralpak AD-H), *n-*hexane:iPrOH 85/15, 0.1 mL/min, λ = 250 nm), *t*_R,maj_ = 12.5 min, *t*_R,min_ = 15.5 min.
